# Thermal Stress and Habitat Loss: Assessing the Vulnerability of *Potamon fluviatile* (Decapoda: Potamidae) to Climate Change in Italy

**DOI:** 10.1002/ece3.74256

**Published:** 2026-09-01

**Authors:** Saman Ghasemian Sorboni, Sharareh Pourebrahim, Jit Ern Chen, Mehrdad Hadipour

**Affiliations:** ^1^ Department of Land, Environment, Agriculture and Forestry (TeSAF) University of Padova Legnaro Italy; ^2^ Jeffry Sachs Center on Sustainable Development Sunway University Selangor Malaysia; ^3^ Sunway Institute for Global Strategy and Competitiveness Sunway University Selangor Malaysia

**Keywords:** climate refugia, ensemble modeling, Mediterranean freshwater crab, range contraction, thermal tolerance

## Abstract

Climate change has the potential to alter the distribution of freshwater species and challenge the effectiveness of existing conservation networks. However, projections of the climatic suitability of *Potamon fluviatile* under recent climate scenarios are lacking for Italy. In this study, we modeled the potential climatic distribution of the species using an ensemble species distribution modeling approach based on multiple future climate projections. Results indicate that temperature conditions during the dry season are an important predictor of the species' large‐scale climatic suitability. Future projections show a contraction of suitable areas, with moderate declines under low‐emission scenarios (SSP1‐2.6) and substantial reductions exceeding 80% by 2070 under high‐emission scenarios (SSP5‐8.5). The models show limited expansion into newly suitable areas, although dispersal processes were not represented. Comparing projected suitability with the current protected area network shows that existing conservation boundaries may not cover future suitable areas. While some large protected areas may experience declines in suitability, some smaller coastal reserves may retain stable climatic conditions. It is important to consider that our findings are based on coarse‐scale climatic predictors and do not account for hydrological processes or fine‐scale habitat characteristics. Our study provides a large‐scale assessment of potential climate‐driven changes in climatic suitability for *P. fluviatile* in Italy. Integrating climatic, hydrological, and ecological processes in future models would improve conservation planning for freshwater species.

## Graphical abstract

Ensemble species distribution models indicate that *Potamon fluviatile* may experience a substantial reduction in climatically suitable habitat in Italy under future climate scenarios, particularly under high‐emission pathways. Comparison with the current protected area network suggests that some existing reserves may lose suitability, while a limited number of smaller areas may remain as potential refugia.

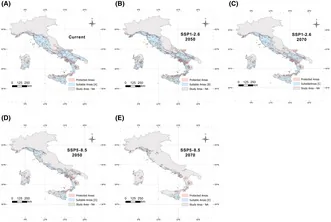

## 1 Introduction

Climate change significantly impacts global freshwater biodiversity by altering precipitation regimes and hydrological cycles (Capon et al. 2021; Reid et al. 2018; Trenberth 2011). In the Mediterranean region, riverine systems are highly vulnerable due to their inherent seasonal flow variability coupled with intense anthropogenic pressure (Gómez‐Navarro et al. 2024). Climate projections for this region indicate an increased frequency of severe droughts and reduced annual discharge, leading to higher rates of stream intermittency (Schneider et al. 2013; Feyen and Dankers 2009). These climatic shifts exacerbate existing anthropogenic stressors, such as dam construction and land‐use alterations, which have severely fragmented these river networks (Andrew and Sauquet 2017; Palmer et al. 2009). Consequently, the compounding effects of thermal stress, flow reduction, and habitat isolation pose an acute risk to the specialized aquatic biodiversity endemic to Mediterranean inland waters (Fernández‐Martínez et al. 2023; Li et al. 2024; Benson et al. 2021; Cramer et al. 2018).

Freshwater crabs serve as critical and keystone species within these riverine networks (Hao et al. 2021). They function as ecosystem engineers, accelerating nutrient cycling through the breakdown of leaf litter and serving as a vital link in the food web between detritus and higher predators (Wang et al. 2023; Xie et al. 2022). However, their physiological and reproductive traits make them highly vulnerable to environmental fluctuation (Lam et al. 2022; Jimenez et al. 2022). As poikilothermic organisms, their metabolism and growth are governed by ambient water temperatures (Azra et al. 2019). Consequently, thermal anomalies can quickly exceed their physiological thresholds, leading to increased mortality (Daunde et al. 2026).

In addition to these physiological constraints, unlike many marine crustaceans that possess a planktonic larval phase to facilitate wide dispersal, freshwater crabs undergo direct development, hatching as miniature adults with very limited mobility (Wu et al. 2010). This low dispersal capacity acts as a trap during rapid environmental change, preventing them from easily migrating to more suitable habitats and contributing to the high extinction risk currently facing the group globally (Cumberlidge et al. 2009). This vulnerability is compounded by the physical structure of riverine systems, which restrict movement to a single dimension along the watercourse. Consequently, simple geographic distance is a poor predictor of connectivity; populations in adjacent but separate catchments remain completely isolated unless connected by extreme flooding events (Daniels et al. 2006).

**FIGURE 1:**
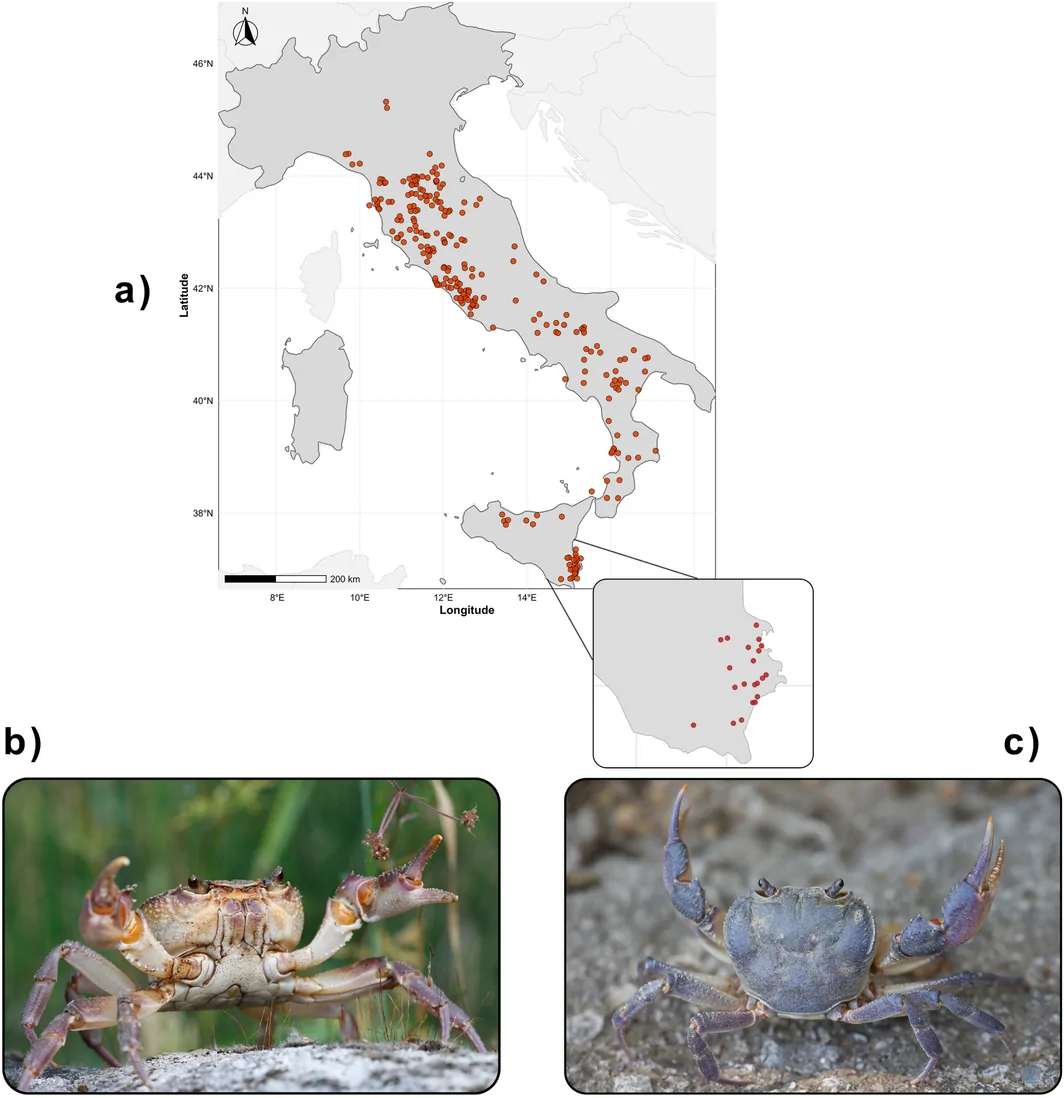
Geographic distribution and morphology of the study organism: (a) geographical distribution of *Potamon fluviatile* occurrence records within the Italian study area used for species distribution modeling; (b, c) photographic representations of adult *P. fluviatile* specimens. Photo credits: (b) Leaper (2020); (c) Helitas (2016) both via iNaturalist, licensed under CC‐BY‐NC 4.0.

In southern Europe, *Potamon fluviatile* (Herbst, 1785) is an endemic freshwater crab that inhabits the fragmented river basins of Italy, the Balkan Peninsula, and smaller islands such as Malta, often existing in isolation (Brandis et al. 2000) (Figure 1b,c). However, its populations are under threat because of climatic changes (Yousefi et al. 2022). Despite these warning signs, there remains a critical lack of detailed projections regarding how the distribution of *P. fluviatile* in Italy will respond to the specific climatic trajectories expected in the coming decades.

To tackle this uncertainty, Species Distribution Models (SDMs) offer an analytical framework for anticipating biodiversity shifts (Franklin 2023; Miller 2010). This study aims to evaluate the potential distribution of *P. fluviatile* in Italy, contrasting its current range with future projections for the years 2050 and 2070. By utilizing two distinct climate pathways, the sustainability‐focused and the fossil‐fuel‐intensive scenarios, this research seeks to quantify the spatial extent of habitat loss or gain, thereby providing the necessary data to inform long‐term conservation strategies for this emblematic freshwater species. Finally, we assess the capacity of the current Italian Protected Area network to buffer these projected shifts, determining whether existing static boundaries will remain effective under dynamic future climatic conditions.

## 2 Methodology

### 2.1 Study Area and Species Occurrences

The study area encompassed the entire territory of Italy. Georeferenced occurrence records for *P. fluviatile* were obtained from the Global Biodiversity Information Facility (GBIF). The initial dataset included 249 records. To remove potentially erroneous and biased records, we cleaned the occurrence data using the R package CoordinateCleaner (Zizka et al. 2019). Occurrence points were further checked against the study area boundaries to ensure geographic consistency. After filtering, a total of 237 spatially independent records were retained for analysis (Figure 1a).

To characterize the environmental background, pseudo‐absence points were generated using the internal background point generator of the *sdm* package (Naimi and Araújo 2016). A total of 237 pseudo‐absences were sampled across the terrestrial extent of the study area utilizing a random geographic selection strategy. To ensure these background points represented spatial conditions distinct from known habitats, a spatial constraint was applied to prevent any pseudo‐absence from overlapping the exact grid cells of the established presence records. This approach successfully maintained a 1:1 presence‐to‐pseudo‐absence ratio (Valavi et al. 2020; Zhang et al. 2019; Barbet‐Massin et al. 2012).

### 2.2 Environmental Predictors

A total of 22 environmental predictors were considered, including 19 bioclimatic variables and three topographic variables. Bioclimatic variables were obtained from WorldClim v2.1 at a spatial resolution of 30 arc‐seconds (~1 km) (Fick and Hijmans 2017), representing biologically meaningful summaries of temperature and precipitation derived from the BIOCLIM framework (Booth et al. 2013; Booth 2025). These data were extracted for current conditions and for two future periods (2041–2060 and 2061–2080) based on five General Circulation Models (GCMs) from CMIP6 (MIROC6, MRI‐ESM2‐0, MPI‐ESM1‐2‐HR, EC‐Earth3‐Veg, and CNRM‐CM6‐1).

Topographic variables (elevation, slope, and aspect) were derived from the same dataset, with slope and aspect calculated in R. To reduce multicollinearity and potential overfitting, a Variance Inflation Factor (VIF) analysis was conducted using the *usdm* package (Naimi et al. 2013), applying a threshold of VIF < 5. The final set of predictors showed reduced collinearity, with pairwise correlations ranging from −0.003 to −0.77 (Table 1).

**TABLE 1:** List of the selected environmental predictor variables retained for the species distribution modeling of *P. fluviatile* after the multicollinearity analysis, along with their corresponding Variance Inflation Factor (VIF) values.

Variable	Abbreviation	VIF	Variable	Abbreviation	VIF
Precipitation Seasonality	Bio15	2.49	Mean Temp. Wettest Quarter	Bio8	2.52
Precip. Warmest Quarter	Bio18	4.33	Mean Temp. Driest Quarter	Bio9	4.63
Precip. Coldest Quarter	Bio19	2.27	Slope	Slope	2.00
Isothermality	Bio3	1.41	Aspect	Aspect	1.02
Temperature Seasonality	Bio4	3.23			

### 2.3 Model Building

Species distribution models were constructed using the sdm package (Naimi and Araújo 2016) within the R statistical environment. To predict the changes, three distinct machine learning algorithms were employed: Random Forest (RF), Support Vector Machine (SVM), and Boosted Regression Trees (BRT). The data were partitioned using cross‐validation method, with 80% of the records allocated for model training and the remaining 20% reserved for testing and validation. Model performance was evaluated using three standard metrics: the Area Under the Receiver Operating Characteristic Curve (AUC; Marmion et al. 2008), the True Skill Statistic (TSS), and Cohen's Kappa (Allouche et al. 2006).

To reduce uncertainty and improve predictive reliability, an ensemble modeling approach was adopted to generate the final suitability maps (Valavi et al. 2021; Araujo and New 2007). This process involved a weighted averaging method, where models were combined based on their performance scores. To ensure the quality of the projections, low‐performing models, defined as those with AUC < 0.7 or TSS < 0.5, were excluded from the ensemble (Khalatbari Limaki et al. 2021). Finally, the maximum sum of sensitivity and specificity (MaxSSS) statistic was utilized (Liu et al. 2013) to determine the optimum threshold for converting continuous probability maps into binary presence/absence maps.

### 2.4 Protected Area Assessment

To evaluate the overlap between climatically suitable areas and conservation zones, spatial data from the World Database on Protected Areas (WDPA; UNEP‐WCMC and IUCN 2025) were used. A two‐step analysis was conducted in ArcGIS Pro. First, protected area polygons were dissolved into a single layer to estimate the total proportion of climatically suitable area falling within the protected network. Second, analyses were repeated using individual protected area units to quantify both the total suitable area and the proportion of each protected area classified as suitable. Suitability maps were intersected with protected area polygons using raster‐based zonal statistics, and pixel counts were converted to area (km^2^).

## 3 Results

### 3.1 Model Performance

**FIGURE 2:**
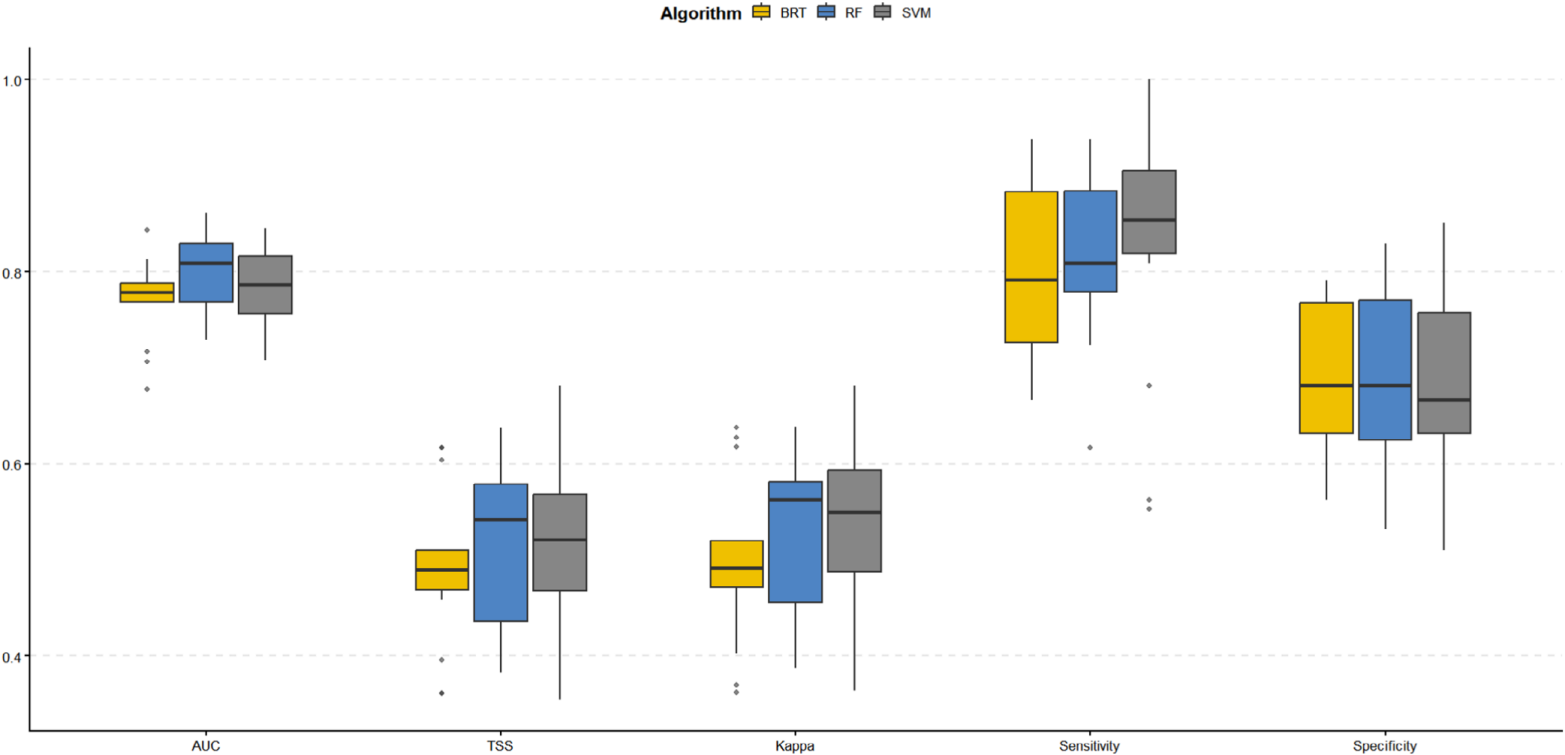
Comparison of predictive performance across the three modeling algorithms (BRT, RF, and SVM). The boxplots summarize the variance in five key evaluation metrics (AUC, TSS, Kappa, Sensitivity, and Specificity) across the model runs, illustrating the median, interquartile range, and outliers for each algorithm.

The ensemble modeling approach yielded good predictive performance across the tested algorithms. Among them, the Random Forest (RF) model showed the highest consistency, achieving a mean AUC of approximately 0.81, followed closely by SVM (0.79) and BRT (0.77). Both TSS and Cohen's Kappa metrics indicated moderate to good model agreement, with mean values ranging from 0.48 to 0.52. The evaluation metrics further showed a distinct performance pattern: models exhibited high sensitivity (mean Se ≈0.85) and moderate specificity (mean Sp ≈0.65) (Figure 2).

### 3.2 Importance of Environmental Predictors

**FIGURE 3:**
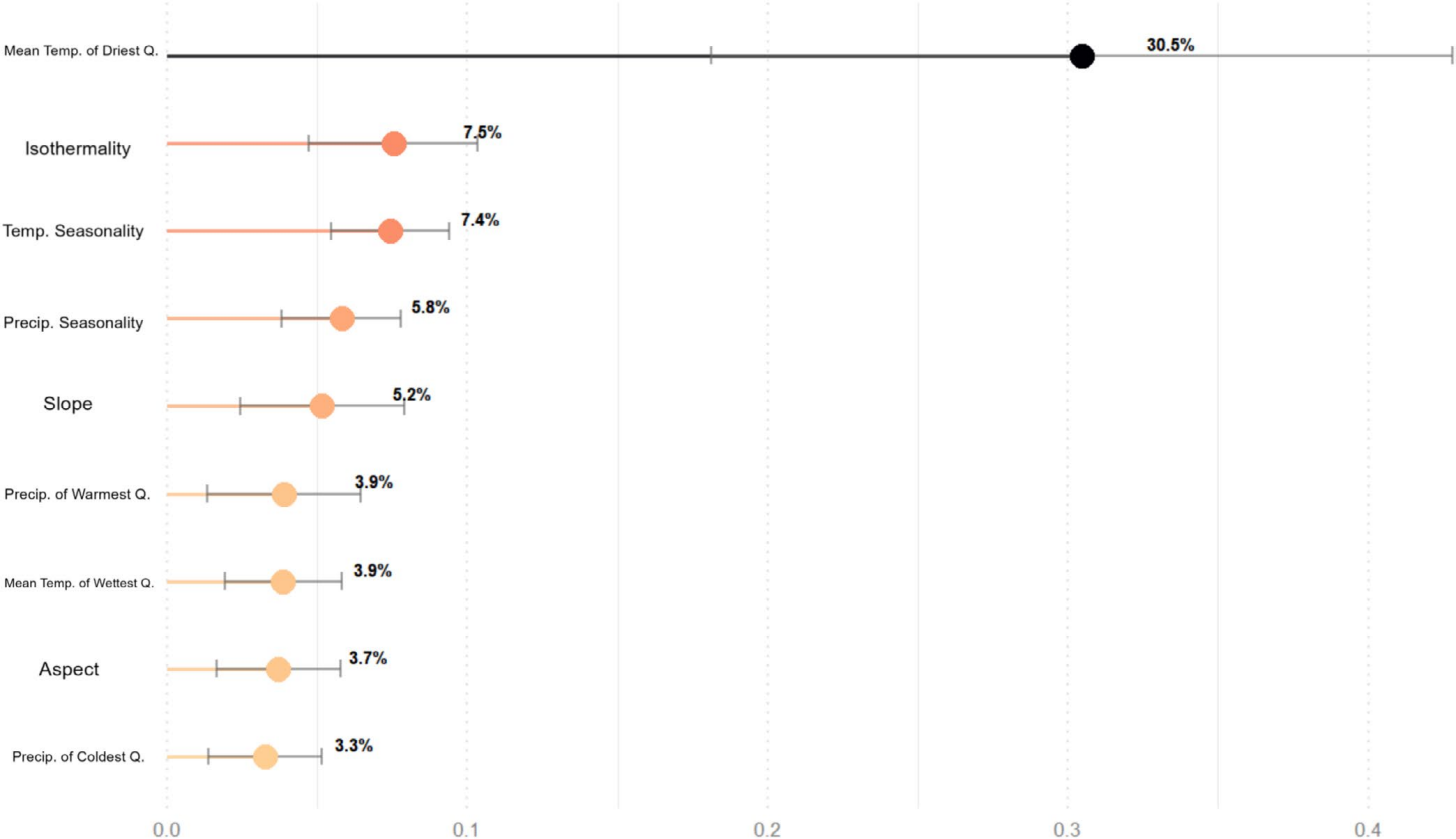
Relative importance of environmental predictors for the *P. fluviatile* ensemble distribution model. Values denote the mean contribution (%) of each variable, highlighting the Mean Temperature of the Driest Quarter (Bio9) as the dominant climatic constraint. Error bars represent the variance across the individual model algorithms.

The analysis of variable importance identified the Mean Temperature of Driest Quarter (Bio9) as the dominant environmental driver for the distribution of *P. fluviatile* in Italy. This variable alone accounted for 30.5% of the model's relative importance (Figure 3). Secondary contributions were observed from Isothermality (Bio3) and Temperature Seasonality (Bio4), which contributed 7.5% and 7.4%, respectively. In contrast, precipitation‐related variables (Bio15, Bio18, Bio19) and topographic features (slope and aspect) played a comparatively minor role in defining the species' spatial suitability.

### 3.3 Prediction Maps and Future Distribution

**FIGURE 4:**
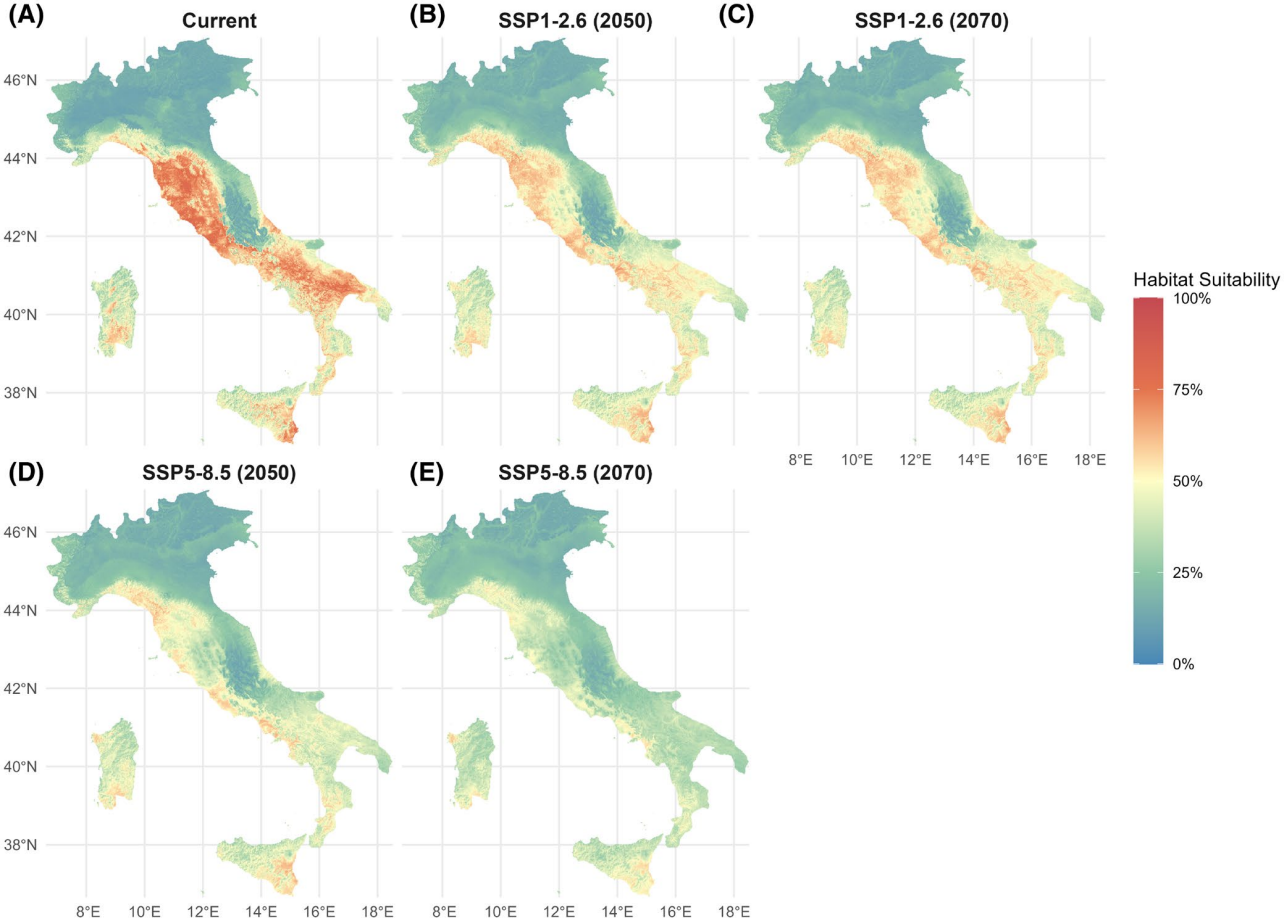
Projected habitat suitability for *P. fluviatile* in Italy across different climatic scenarios. The maps display the ensemble probability of occurrence, derived from RF, SVM, and BRT algorithms, on a continuous scale from 0 (unsuitable) to 1 (optimal suitability). Panels represent: (A) Current climatic conditions; (B, C) Projections for the years 2050 and 2070 under the low‐emission scenario (SSP1‐2.6); and (D, E) Projections for the years 2050 and 2070 under the high‐emission scenario (SSP5‐8.5). Note that these maps represent broad‐scale macro‐climatic suitability across the terrestrial landscape; the species' realized habitat is restricted to river networks within these highly suitable zones.

The distribution models indicate that under current climatic conditions, the climatic envelope suitable for *P. fluviatile* covers approximately 114,482 km^2^ of the study area. This value represents macro‐climatic suitability across the landscape, whereas the species' actual realized habitat is confined to riverine networks within these climatically suitable zones. However, future projections show a consistent decrease in suitable climatic space across all emission scenarios (Figure 4).

Under the SSP1‐2.6 scenario, suitable habitat is projected to decrease by approximately 12.7% (to 99,913 km^2^) by 2050, increasing to a 17.9% reduction (93,854 km^2^) by 2070 relative to the baseline. In contrast, the SSP5‐8.5 scenario predicts larger reductions, with the total suitable area decreasing by 47.6% (to 60,246 km^2^) in 2050 and by 80.8% (to 22,238 km^2^) by 2070.

**FIGURE 5:**
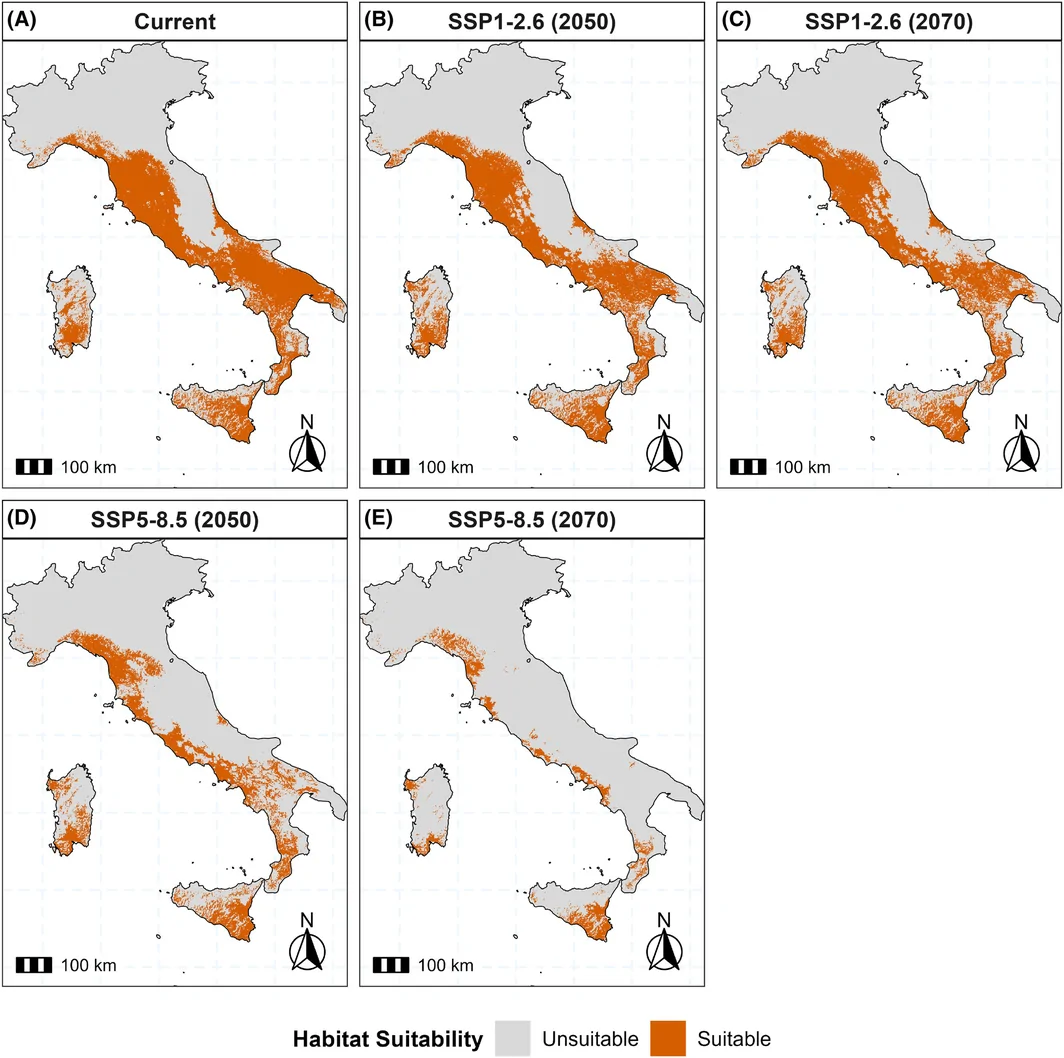
Projected range contraction of *P. fluviatile* in Italy under future climate scenarios. The maps display ensemble binary habitat suitability, derived by applying the maximum sum of sensitivity and specificity (MaxSSS) threshold to the continuous probabilities shown in Figure 4. Orange indicates suitable climatic space and gray indicates unsuitable areas. “Range contraction” refers to the net loss of suitable area (km^2^) relative to the current distribution. Panels show: (A) Current distribution; (B, C) Future projections for 2050 and 2070 under the sustainable SSP1‐2.6 scenario; and (D, E) Future projections for 2050 and 2070 under the high‐emission SSP5‐8.5 scenario. Similar to Figure 4, suitable areas represent the macro‐climatic envelope, not explicit riverine pathways.

Spatially, these changes occur primarily as a contraction of the species' current climatic range rather than a displacement to newly suitable areas. Consequently, the proportion of stable climatic conditions, areas that remain suitable across timeframes, decreases. Across all modeled scenarios, the net change in suitable area is consistently negative. The extent of climatic suitability loss increases with both time and higher radiative forcing, with the largest reduction observed under the SSP5‐8.5 scenario in 2070 (Figure 5).

### 3.4 Protected Area Network Coverage

Under current climatic conditions, approximately 21.5% of the modeled suitable habitat for *P. fluviatile* overlaps with the national protected area network. The National Park of Cilento, Vallo di Diano e Alburni contains the largest extent of suitable habitat (approximately 1526 km^2^), followed by the Pollino (1297 km^2^) and Murgia Alta (1254 km^2^) National Parks. In terms of proportional coverage, Murgia Alta exhibits the highest suitability among the largest protected areas, with 85.1% of its total area classified as suitable. Other protected areas showing high fractional suitability (> 80%) include the Comprensorio Tolfetano‐Cerite‐Manziate (90.4%) and the Alta Murgia National Park (82.2%).

**FIGURE 6:**
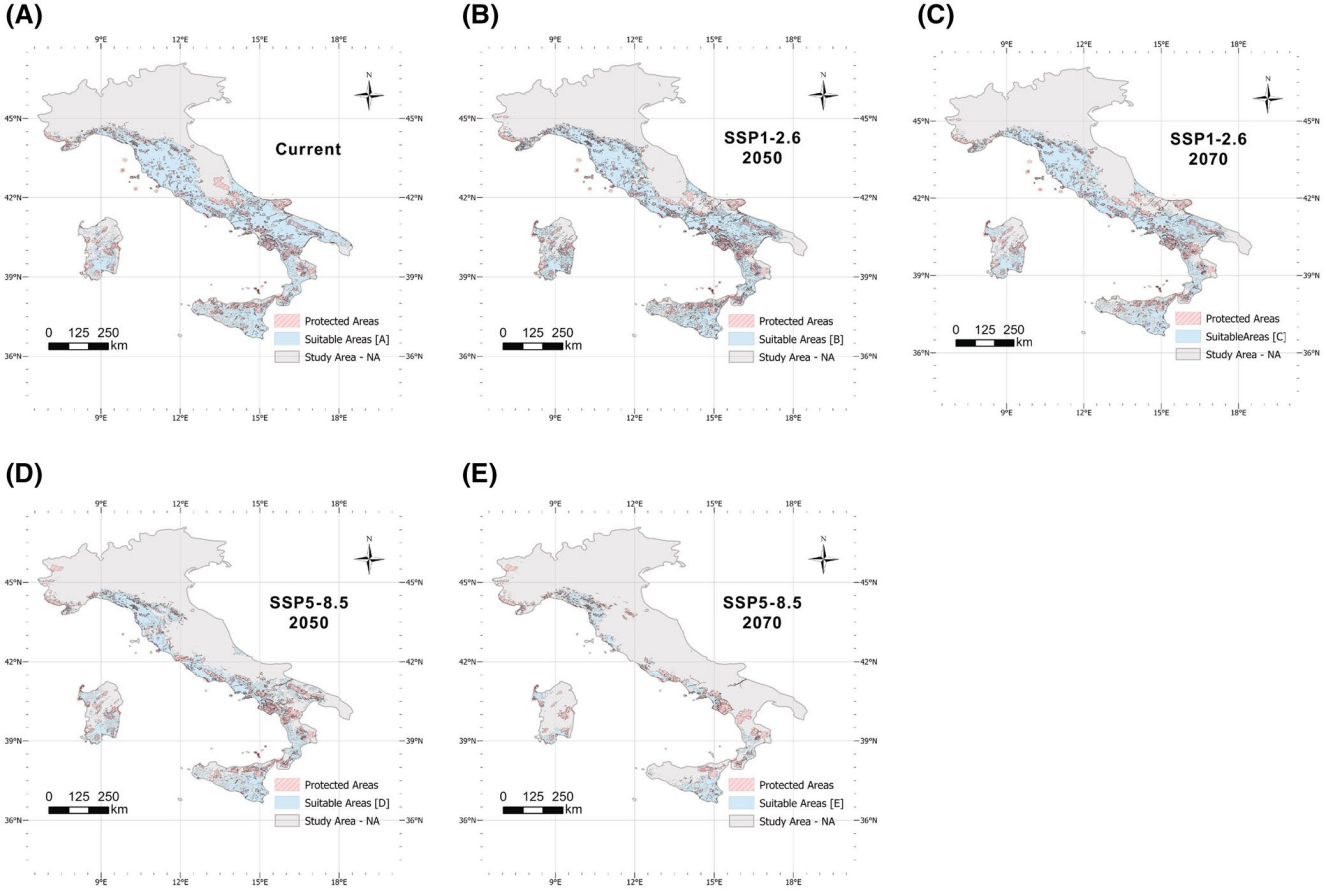
Spatial overlap between the Italian terrestrial protected area network (red hatched areas) and the projected suitable climatic habitat (blue) for *P. fluviatile* . Panels represent: (A) current climatic conditions; (B, C) future projections for 2050 and 2070 under the SSP1‐2.6 scenario; and (D, E) future projections for 2050 and 2070 under the SSP5‐8.5 scenario.

Future projections indicate a reduction in protected area coverage across both emission scenarios. Under the SSP1‐2.6 scenario, the proportion of suitable habitat within the protected network decreases to 19.5% by 2050 and 19.2% by 2070. The hierarchical ranking of protected areas remains largely consistent with the baseline; Cilento and Murgia Alta remain the top‐ranked areas for total suitable habitat. Suitability within the Pollino National Park declines from a baseline of 49.4% to 43.6% by the period 2061–2080. Under the SSP5‐8.5 scenario, the total proportion of protected suitable habitat declines to 20.0% by 2050 and 15.6% by 2070. By the 2061–2080 period, the suitable habitat area within Cilento decreases to 12.4% coverage (~266 km^2^), though it remains the top‐ranked area. Aspromonte National Park becomes the second‐ranked area with approximately 231 km^2^ of suitable habitat (35.5% coverage). While suitable area within large national parks decreases, specific smaller reserves such as Torre Manfria, Biviere e Piana di Gela and Migliarino, San Rossore e Massaciuccoli maintain suitability percentages exceeding 87% and 93%, respectively (Figure 6).

## 4 Discussion

The ensemble modeling approach utilized in this study provided a reasonable approximation of the species' climatic niche, with Random Forest (RF) and Support Vector Machine (SVM) algorithms showing relatively consistent performance (AUC > 0.8). However, given the use of cross‐validation and potential spatial autocorrelation in occurrence records, these evaluation metrics should be interpreted with caution, as they may overestimate predictive performance. The models exhibited high sensitivity (Se ≈0.85), indicating a strong ability to capture areas of potential climatic suitability for *P. fluviatile* (McPherson et al. 2004). In contrast, the lower specificity (Sp ≈0.65) is consistent with common patterns in species distribution modeling (Olden et al. 2002), where species do not occupy all climatically suitable areas due to factors such as historical dispersal limitations (Blinkhorn et al. 2025) or biotic interactions, including competition and predation (Siemens et al. 2023). As is inherent to correlative SDMs, these outputs represent potential climatic suitability rather than realized distribution, and should therefore be interpreted within this context.

A key result of this study is the prominent contribution of Mean Temperature of the Driest Quarter (Bio9), which accounted for over 30% of model importance. This finding suggests that the large‐scale distribution of *P. fluviatile* is strongly associated with temperature conditions during the dry season. While this pattern may reflect vulnerability to combined heat and seasonal aridity, this study was specifically designed to capture broad, macro‐climatic impacts based on standardized IPCC scenarios. Therefore, the model relies on atmospheric climate variables and does not incorporate fine‐scale hydrological variables (such as permanent watercourses, water temperature, or oxygenation). Consequently, any linkage between high atmospheric temperatures and reduced stream flow conditions should be considered inferred based on general ecological understanding of Mediterranean freshwater systems (Carosi 2022; Mulholland et al. 1997), rather than directly tested in this study. As a poikilothermic organism, *P. fluviatile* similar to *P. ibericum*, depends on ambient thermal conditions for metabolic regulation (Ghasemian Sorboni et al. 2025), and elevated temperatures during the dry season may represent a key environmental constraint at broad spatial scales. In contrast, topographic predictors (slope and aspect) showed relatively low importance, suggesting that although the species likely utilizes local microhabitats (e.g., shaded riverbanks) to buffer thermal extremes (Anger et al. 2007), such fine‐scale processes are not fully captured by the coarse spatial resolution of the predictors used here (Kawaida et al. 2017). While large‐scale atmospheric variables like the Mean Temperature of the Driest Quarter serve as essential proxies for regional thermal constraints, they do not perfectly mirror the actual water temperatures experienced by this benthic crab. Consequently, local aquatic microclimates may be more thermally buffered than the macro‐climatic grids suggest, allowing populations to persist in localized refugia even as the broader climatic envelope becomes unsuitable.

Consistent with the importance of dry‐season temperature, future projections indicate a decline in climatic suitability for *P. fluviatile* across Italy. While the current potential distribution is relatively widespread, all scenarios project a progressive contraction of suitable climatic space. Under the SSP1‐2.6 pathway, projected habitat loss remains moderate (−12.7% to −17.9%), suggesting that lower‐emission trajectories may partially mitigate the extent of contraction. In contrast, the SSP5‐8.5 scenario indicates a much stronger reduction, with over 80% of currently suitable areas projected to fall outside the species' present climatic envelope by 2070. Such patterns are broadly consistent with reported trends for freshwater taxa in Mediterranean regions, where increasing temperatures and altered climatic regimes are expected to drive range contractions (Heino et al. 2009). However, it is important to consider that these projections represent changes in climatic suitability and do not account for dispersal processes, habitat connectivity, or local environmental conditions. Although freshwater crabs are generally constrained to riverine systems and are characterized by limited dispersal capacity (Sun et al. 2020), the extent to which *P. fluviatile* can track shifting climatic conditions remains uncertain.

Our results indicate a progressive contraction of climatically suitable areas rather than a clear shift toward newly suitable regions. The analysis of range change metrics suggests that potential expansion into new areas is limited (approximately 3.4%–9%) relative to the projected loss of currently suitable climatic space. This imbalance may indicate a reduced capacity for natural range expansion; however, dispersal processes were not modeled. Although *P. fluviatile* is characterized by direct development and generally low dispersal ability, and is further constrained by the one‐dimensional dispersal pathways of isolated river networks, the extent to which it can track shifting climatic conditions remains uncertain within the scope of this study.

The magnitude of the projected decline under the SSP5‐8.5 scenario raises potential conservation concerns. If regional climatic conditions follow high‐emission trajectories, *P. fluviatile* could face increasing risk, although any reassessment of its conservation status would require integration of additional ecological and demographic data beyond climatic suitability alone. The fragmentation of river systems in Italy (Billi and Fazzini 2017), together with projected changes in seasonal temperature regimes, may contribute to increased isolation of populations, especially where local environmental conditions diverge from historically suitable climates (Titeux et al. 2019; Robertson and Hutto 2006).

The projected reduction in macro‐climatic suitability for *P. fluviatile* aligns with modeling efforts for other *Potamon* species across the Mediterranean and Near East. Endemic freshwater crabs in these regions show comparable responses to future climate projections. For example, distribution models suggest that the macro‐climatic suitability for species such as *P. hippocratis* and *P. pelops* could decline by 75% to 100% under future scenarios (Yousefi et al. 2022). Similarly, more widely distributed species like *P. ibericum* exhibit high sensitivity to temperature seasonality and the mean temperature of the driest quarter. Projections for *P. ibericum* indicate up to a 96% reduction in its suitable macro‐climatic envelope by 2070 under the SSP5‐8.5 scenario, with potential shifts toward higher elevations (Ghasemian Sorboni et al. 2025). These shared patterns indicate that the climatic constraints identified for *P. fluviatile* are consistent across the genus within these fragmented drainage basins.

The evaluation of the Italian protected area network suggests a potential mismatch between current conservation boundaries and projected patterns of climatic suitability. Approximately 21.5% of the currently suitable climatic space overlaps with the protected area network; however, several large National Parks, including Cilento and Pollino, are projected to experience declines in climatic suitability under high‐emission scenarios by 2070. Existing protected areas, while essential for conservation, may not fully align with future patterns of climatic suitability (Cui et al. 2025; Hannah 2008). It is important to emphasize that this analysis is based on coarse‐resolution climatic data and terrestrial grid cells, and therefore represents climatic overlap rather than a direct measure of protected riverine habitat.

Conversely, some smaller coastal reserves appear to maintain relatively high levels of climatic suitability across scenarios, suggesting that they may function as areas of relatively stable macro‐climatic conditions. Sites such as Torre Manfria and San Rossore are projected to retain high suitability values (> 85%) despite overall range contraction. While these areas may represent potential zones of persistence, it should be noted that the model does not incorporate fine‐scale hydrological or habitat characteristics. Therefore, any inference regarding their role as refugia should be considered tentative and subject to further investigation using more detailed environmental data.

From a management perspective, conservation strategies may benefit from considering areas that are projected to retain relatively stable climatic conditions. However, potential actions such as riparian restoration or environmental flow management address processes that are not represented in the current modeling framework and should therefore be viewed as complementary considerations rather than direct outcomes of the model. Similarly, assisted colonization (translocation) may be discussed in the context of extreme future scenarios, but such interventions remain highly uncertain and require careful evaluation of ecological risks, including impacts on recipient ecosystems and disease transmission (Gardner and Bullock 2025).

Finally, several limitations inherent to the Species Distribution Modeling (SDM) framework used in this study must be acknowledged. Occurrence data derived from GBIF are likely affected by spatial sampling bias, often favoring accessible or well‐surveyed areas (Beck et al. 2014; Sorboni et al. 2026), potentially underrepresenting populations in less‐sampled regions (Lin et al. 2022). Also, the models characterize aspects of the species' climatic niche rather than its realized distribution (Beery et al. 2021), and are specifically designed to evaluate large‐scale vulnerability under standardized IPCC macro‐climatic scenarios (IPCC 2022). As such, they do not incorporate critical local parameters such as water temperature, oxygenation, biotic interactions, or anthropogenic dispersal barriers (e.g., dams or intermittent river sections), or local anthropogenic stressors such as water pollution (Boyer et al. 2023).

Projections were derived from multiple algorithms and climate models, but uncertainty among these sources was not quantified; therefore, reported values of habitat change should be interpreted as approximate estimates rather than precise predictions. Finally, because model calibration was restricted to occurrence data from Italy, the results reflect a regional portion of the species' niche rather than its full environmental tolerance.

Given these limitations, and considering the importance of local hydrological and habitat conditions for freshwater species, the actual area available for occupancy is likely more restricted than suggested by coarse‐scale climatic suitability maps. Accordingly, areas identified as potential future “gains” should be interpreted cautiously as climatically suitable under broad conditions, rather than as guaranteed habitats for future colonization (Liepe et al. 2022).

## 5 Conclusion

Our study provides a broad assessment of *P. fluviatile* potential climate change impacts under CMIP6 emission scenarios in Italy. The results suggest that temperature conditions during the summer dry season are a key predictor of the species' large‐scale climatic suitability. Future projections indicate large reductions in suitable climatic space, particularly under high‐emission scenarios.

The projected contraction of suitable areas, combined with the fragmented nature of river systems, may reduce the ability of *P. fluviatile* to track shifting climatic conditions; however, dispersal processes and local habitat dynamics were not modeled and remain important sources of uncertainty. Also, the comparison with the current protected area network suggests that existing conservation boundaries may not fully align with future patterns of climatic suitability, although this assessment is based on macro‐scale climatic overlap rather than direct representation of aquatic habitats.

Our findings support considering climate‐driven changes in large‐scale suitability when evaluating conservation strategies. Future research integrating hydrological variables, dispersal processes, and fine‐scale habitat characteristics would help refine these projections and support management decisions for freshwater species under climate change.

## Author Contributions

**Saman Ghasemian Sorboni:** conceptualization (equal), data curation (equal), formal analysis (equal), methodology (equal), software (equal), writing – original draft (equal). **Sharareh Pourebrahim:** conceptualization (equal), data curation (equal), formal analysis (equal), writing – original draft (equal), writing – review and editing (equal). **Jit Ern Chen:** funding acquisition (lead), project administration (equal), resources (equal). **Mehrdad Hadipour:** methodology (equal), supervision (equal), writing – original draft (equal).

## Funding

This work was supported by Sunway University, SUR‐JSC‐SSERV‐2024‐13 Sunway Group ESG grant.

## Conflicts of Interest

The authors declare no conflicts of interest.

## Data Availability

The data that underpin the findings of this study are openly available in https://zenodo.org/ at [https://doi.org/10.5281/zenodo.19634877]. Primary occurrence data were retrieved from the Global Biodiversity Information Facility (GBIF) [https://doi.org/10.15468/39omei accessed via GBIF.org on 2025‐07‐11]. Environmental predictors were obtained from the WorldClim v2.1 database (https://www.worldclim.org/data/cmip6/cmip6climate.html), and protected area boundaries were sourced from the World Database on Protected Areas (WDPA) [www.protectedplanet.net].
